# A Rare Subepithelial Lesion of the Proximal Colon Evaluated Using Miniprobe Endoscopic Ultrasonography: A Case Report

**DOI:** 10.7759/cureus.79793

**Published:** 2025-02-27

**Authors:** Hidetaka Hamamoto, Tomoki Matsuda, Mareyuki Endo

**Affiliations:** 1 Department of Gastroenterology, Sendai Kousei Hospital, Sendai, JPN; 2 Department of Surgical Pathology, Sendai Kousei Hospital, Sendai, JPN

**Keywords:** colonic anisakiasis, colonoscopy, colon subepithelial lesion, endoscopic submucosal dissection, endoscopic ultrasonography, miniprobe endoscopic ultrasonography

## Abstract

Preoperative diagnosis of submucosal lesions in the colon can be challenging. Total colonoscopy comprising miniprobe endoscopic ultrasonography (mEUS) revealed a 5-mm submucosal lesion in the transverse colon as a hypoechoic area primarily within the submucosa in our patient. However, a definitive diagnosis could not be made after evaluating the biopsy results. Based on the mEUS findings, adhesion to the muscularis was localized; therefore, endoscopic submucosal dissection was performed. A postoperative pathology examination revealed a parasite-like structure at the center of the necrotic tissue that was diagnosed as a colorectal-type necrotic nodule. Diagnostic EUS should be considered for such cases, and mEUS is useful for assessing the safety of specimen collection. This technique is especially valuable for diagnosing colorectal submucosal lesions in the proximal colon, where other diagnostic modalities are limited, and may contribute to safer endoscopic mucosal resection and endoscopic submucosal dissection.

## Introduction

The consumption of raw or undercooked food containing Anisakis larvae can lead to infections in humans. However, humans are accidental hosts of such larvae, and the development of anisakiasis in humans is rare. Anisakis larvae usually embed in the gastric or intestinal mucosa and eventually die, but, in rare cases, they may cause intestinal perforation or anaphylaxis. Anisakiasis is a condition that is caused when larvae of the Anisakidae family invade the human digestive tract, resulting in acute gastric inflammation; therefore, colonic anisakiasis is rare [1,2]. When no worm bodies are identified on the luminal side, intestinal anisakiasis should be considered as part of the differential diagnosis for submucosal lesions (SELs) of the large intestine [3-7]. Because the diagnostic accuracy of endoscopic ultrasonography (EUS) for colonic SELs is only 48%, it is insufficient for determining the definitive diagnosis. Therefore, histological confirmation should be performed whenever possible [8]. If a SEL is present in the right-sided colon, then performing an EUS-guided needle biopsy to obtain diagnostic specimens is challenging. Therefore, it is important to identify the main layer of the colon wall in advance to select an appropriate alternative method. Our case comprised asymptomatic intestinal anisakiasis presenting as an SEL in the transverse colon with necrotic nodules that was successfully diagnosed and treated by performing endoscopic submucosal dissection (ESD) with a traction device.

## Case presentation

**Figure 1:**
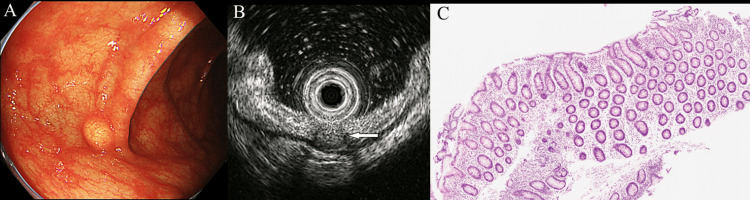
Endoscopic findings. (A) The subepithelial lesion (SEL) appeared yellow in the transverse colon and did not exhibit epithelial changes. (B) Using miniprobe endoscopic ultrasonography (mEUS), the lesion was characterized as a hypoechoic mass located in the deep submucosal layer (arrow) and superficial muscularis propria with a marginal hypoechoic zone. (C) A deep biopsy was performed from the apex of the SEL; however, a definitive diagnosis was not determined.

A 61-year-old woman was referred to our hospital for the evaluation and treatment of a 5-mm SEL in the transverse colon after total colonoscopy. She had a history of appendectomy that was performed because of appendicitis; however, she did not have a history of malignant disease. Itopride hydrochloride, famotidine, and oxethazine were administered for reflux esophagitis. The SEL appeared yellow in the transverse colon, and no epithelial changes were observed during endoscopy (Olympus PCF-H290ZI; Olympus, Tokyo, Japan) (Figure 1A). Using miniprobe EUS (mEUS) (20 MHz; Olympus UM-3R; Olympus, Tokyo, Japan), the lesion was characterized as a hypoechoic mass with a marginal hypoechoic zone in the deep submucosal layer and superficial muscularis propria. The mass was in partial contact with the inner muscularis propria (Figure 1B). A deep biopsy was performed from the apex of the SEL; however, a definitive diagnosis was not determined (Figure 1C).

**Figure 2:**
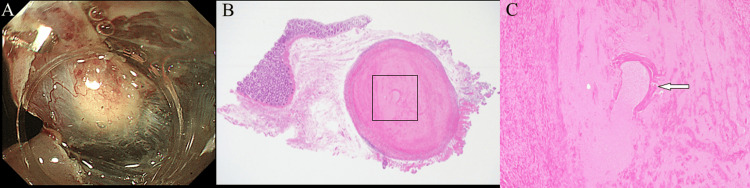
Histological diagnosis. (A) A colorectal subepithelial lesion (SEL) was resected by performing endoscopic submucosal dissection (ESD) with traction using an S-O clip. (B) Hematoxylin and eosin (H&E) staining of the resected specimen. The SEL was considered a granulomatous change. (C) A parasite-like structure was found in the center of the lesion (arrow points to the square area shown in B; original magnification ×200).

mEUS revealed a hypoechoic area with endoscopic morphology and color suggestive of a granular cell tumor or neuroendocrine tumor. Although the contact area between the SEL and the muscularis propria was small, endoscopic submucosal resection with a ligation device is associated with a risk of perforation. Additionally, an endoscopic incisional biopsy may cause scarring after tissue sampling, thus making subsequent endoscopic resection challenging. Therefore, we opted to perform diagnostic treatment using ESD while directly visualizing the SEL and submucosa. ESD with traction was performed using an S-O clip (ZEON Medical, Tokyo, Japan). A white mass with a predominantly submucosal component was observed intraoperatively (Figure 2A). A postoperative pathology examination revealed a parasite-like structure in the center of the mass that was considered a granulomatous change secondary to the presence of a worm, and anisakiasis was suspected because of its frequency in Japan (Figures 2B, 2C). Additional pathology evaluations of the central core were performed; however, necrotic Anisakis nematodes and Y-shaped lateral epidermal cords were not identified. After surgery, we reviewed the patient’s eating habits and found that, like many Japanese people, she regularly ate raw fish.

## Discussion

The exact prevalence of SELs detected by colonoscopy remains unclear [9,10]. However, the results of qualitative assessments including EUS have been reported [10-13]. The diagnostic accuracy of EUS alone is approximately 50% for gastrointestinal SELs in the third and fourth layers of the stomach and colon [8]. Parasitic infections and necrotic nodules comprise 8.6% and 3.8% of all incidental colonic SEL cases, respectively. Most SELs with hard elasticity and no mucosal surface changes are benign lesions, and only 10.7% of cases have malignant potential [13]. Therefore, it is crucial to establish an appropriate treatment plan and avoid overly invasive diagnostic procedures. However, in the proximal colon, the application of tissue diagnosis techniques, such as EUS-guided fine-needle aspiration, can be challenging. A safer and more reliable method of histologically diagnosing SELs in the proximal colon is required. For colorectal tumors, EUS is sometimes used to assess the adhesion between the tumor and muscularis propria to predict the difficulty of ESD. In our case, the main component of the SEL was located in the third layer, and adhesion to the fourth layer was observed. No previous reports have discussed the safe performance of ESD for patients with adhesions between the SEL and muscularis propria when the extent of adhesions is sufficiently narrow. However, ESD is not recommended for early gastric cancers when the third layer is interrupted over an area larger than 5 mm [14]. ESD was performed for this case because the extent of third-layer interruption was less than 4 mm. Although resection was challenging, we successfully performed diagnostic ESD without any issues by utilizing the traction method with an S-O clip for the SEL in our patient. The usefulness of S-O clips has been demonstrated in the stomach [15] and duodenum [16]. Additionally, their effectiveness in the large intestine has been recognized [17].

The specimen obtained from our patient appeared to have the characteristics of a previously reported submucosal necrotic nodule [18]. Approximately 10% of submucosal necrotic nodules are composed of unidentified foreign bodies. An additional pathological evaluation of our case was conducted to detect the presence of a central necrotic Anisakis simplex nematode and Y-shaped lateral epidermal cords; however, these were not identified. Consequently, we concluded that the final diagnosis was necrotic nodules of the colon likely caused by anisakiasis.

Anisakiasis is a parasitic nematode infection that occurs in humans through the consumption of raw or contaminated seafood such as herring, squid, and anchovies [19]. The incidence of this disease is increasing globally [20]. Anisakiasis primarily affects the stomach and causes symptoms such as upper abdominal pain; however, it rarely involves the large intestine [1,2], and an endoscopic diagnosis can be challenging when the worm is not present in the lumen. The clinicopathological features of the reported cases are presented in Table 1 [3-7].

**Table 1:** A brief literature review of reported cases of anisakiasis of the colon that demonstrated the morphology of submucosal lesions without a worm body in the lumen. EMR, endoscopic mucosal resection; ESD, endoscopic submucosal dissection; EUS, endoscopic ultrasonography; F, female; M, male.

	Sex	Age (years)	Symptoms	Location	Lesion size (mm)	EUS	Treatment	Pathological findings
Moschella et al. [3]	F	37	Abdominal pain	Cecum	60	-	Surgery	Cuticle and renette cells
Herranz-Bachiller et al. [4]	F	47	Rectal bleeding	-	-	-	EMR	Eosinophilic abscesses
Hernandez-Prera et al. [5]	F	45	Anemia, rectal bleeding	Sigmoid colon	25	-	Surgery	Cuticle and Y-shaped lateral cords
Pons et al. [6]	F	54	-	Ascending colon	12	-	EMR	Y-shaped lateral cords and eosinophilic abscesses
Martínez-Acitores et al. [7]	M	61	-	Ascending colon	10	-	ESD	Remains of a parasitic structure and the presence of eosinophilic abscesses
Present case	F	61	-	Transverse colon	5	+	ESD	Remains of a parasitic structure and eosinophilic abscesses

The cases presented in Table 1 were diagnosed after procedures such as endoscopic mucosal resection (EMR) [4,6], ESD [7], and surgical resection [3,5] were performed. However, only our patient underwent an mEUS examination prior to endoscopic resection and surgery.

## Conclusions

The postoperative pathology of this case revealed a parasite-like structure at the center of the mass that led to the suspicion of intestinal anisakiasis based on its prevalence in Japan. Because of the benign nature of this condition, mEUS played a crucial role in selecting diagnostic and therapeutic approaches with minimal invasiveness. During mEUS, the parasitic granuloma appeared as a hypoechoic mass located in the deep submucosal and superficial muscularis propria layers with hypoechoic areas at the margins.

mEUS can provide valuable information regarding cases of colorectal SELs, particularly in the proximal colon, by aiding in the selection of appropriate endoscopic approaches, such as EMR and ESD, as well as safe diagnostic and therapeutic options. Furthermore, the use of mEUS may help prevent adverse events such as bowel perforation.
